# American Academy of Dental Science

**Published:** 1902-01

**Authors:** 

**Affiliations:** American Academy of Dental Science


### Reports of Society Meetings.

### AMERICAN ACADEMY OF DENTAL SCIENCE.

The regular monthly meeting of the American Academy of Dental Science was held at Young's Hotel, Boston, Mass., on Wednesday evening, October 2, 1901, at six o'clock, the President, V. C. Pond, in the chair.

A paper was read by Dr. E. K. Wedelstaedt, of St. Paul, Minn., subject, "Faulty Environment."

(For Dr. Wedelstaedt's paper, see page 1.)

### DISCUSSION.

*President Pond.*—Gentlemen, you have listened to this very interesting paper. This is a subject that we have heard discussed a great many times; many of you have expressed your opinions on it upon previous occasions, and I have no doubt many of you would like to do so again, especially with the new light we have received. The subject is open for discussion.

*Dr. Bogue.*—I have not a new idea, not one; unless it be to offer to our friend, Dr. Wedelstaedt, my condolences for the section of country he lives in. The specimens that he has been kind enough to show us here to-night lead me to sympathize most heartily with him. I do not wonder at all that he should have been moved with the holy anger that he has expressed here this evening. If that is the kind of contour filling they have up their way, I am sorry for them. In regard to Dr. Wedelstaedt's paper, I have no words of criticism to offer.

But, gentlemen, Dr. Wedelstaedt left out something. When he talks to us about environment, and gives us to understand that projecting and rough bodies of amalgam are going to lead to or cause an environment that must result disastrously to the patient, I say "Amen" to that; also, when a tooth is extracted from the mouth, wherever that extraction may have occurred, there will be faulty contact. And this I say, notwithstanding Dr. Wedelstaedt's assertion (and he is an authority on faulty environment) that if we would but fill four teeth side by side in the same mouth, two of them after the manner of Dr. Beck, using means for prevention, and two not, we should in a short time learn what prevention means, and adopt it.

Dr. Wedelstaedt will, I am sure, forgive me if I repeat, as well as I can from memory, a few words that I dictated to my amanuensis, over a patient, not many weeks since. "Mrs. B., aged fifty-four or fifty-five; right lower third molar and second molar; proximal cavities filled with amalgam; posterior cavity of third molar also filled with amalgam to the extent of one-fifth of entire crown; the proximal cavities in the second and third molars did not extend to the grinding surfaces, and the cavities did not extend to the gingival margin." But the teeth had been thoroughly prepared, and were as thoroughly contoured as I knew how to do it. The adjoining teeth, the second permanent molar, and the second bicuspid (for the first molars had all been extracted in childhood), were treated to extension in 1877 (month of May, I believe); they were treated to extension, for the delay was extensive, and were filled with gold, in my most artistic manner. Twenty-four years have passed; the posterior amalgam filled teeth are to-day in as good condition as I could imagine, excepting that they have needed a little patching, done in a few moments. The second molar, anterior proximal, and the second bicuspid, posterior proximal cavity, those two gold fillings which touched, after about seven years needed further attention. The decayed parts were cut out as well as I could cut them out, and were patched with amalgam; the gold was left where it was. Two years ago I needed to repair them at the lingual and near the grinding surfaces again.

Now, I am perfectly well aware that I lay myself open to the accusation of having been faulty in my work: I should not dare say I was not. Twenty-four years is a good long time. But the same hand that filled those faulty ones filled also the back ones, the third and second molars, and those have lasted the whole twenty-four years, in the same mouth, and as nearly as possible the same surroundings.

Now, what is the matter? I have no hesitation, gentlemen,—leaving out of the question my own faulty manipulation,—I have no hesitation in saying that the extraction of those first permanent molars for that patient, which necessitated the wedging apart of all the posterior teeth, was very largely to blame for such faulty environment as led to a recurrence of decay time after time. And I also felt very much like advocating thorough cleanliness, by whatever method, and the careful instruction in the methods of cleanliness by every operator to every patient.

*Dr. Fillebrown.*—The gentleman has given us an excellent paper. I have been somewhat familiar with his methods for some years, and my feeling has been that he carries his practice to rather an extreme. I would hardly think it best, in my own practice, to take a cuspid tooth with a medium cavity on the distal side and cut it away so as to have a quarter of it actually a gold filling, when it might have been done without the filling showing at all. But the main proposition is right, that you are generally to cut away the wall of the cavity so that the line of contact of the filling and the tooth-substance shall not rest in contact with any other surface; because, if it does, it will hold a little line of moisture there in a way that will lead to decomposition; and it is hard for the most careful person to keep the surface entirely free.

The point made by my friend at my right is good,—that you must be cleanly. And, secondly, whatever work is done, it must be made as largely as possible self-cleanly.

There is one other idea that the gentleman proposes that I think needs to be guarded against a little. Evils resulting from the operation have, in many cases that have come under my observation, been of greater importance than prevention of recurrence of decay. I believe that where Riggs disease appears on one or two teeth in the mouth, or in different localities, a very large proportion are the result of too severe separation of teeth. It is not natural. The question of how much we shall separate depends altogether upon the person's skill in placing the filling: it is not necessary to have a very large space. Certainly, any one can fill solidly against a matrix, can equally well fill against the opposing surface of a proximal cavity. When that shall have been filled in, and a thin burnisher (not a quarter part as thick as these trimmers that are sent around here) passed between, it would give a good surface to finish, and by that time the teeth can be easily separated sufficiently to give an opportunity to protect the filling, and not do violence to nature by a wide separation.

I wish to say one word, too, in regard to the Arthur separation, so called. It has been condemned in unmeasured terms here to-night. I will say that for nearly twoscore years I have practised that plan, especially upon the anterior teeth, and I have yet to see the first case of recurrence of decay on account of that method of treatment. Some of my fillings have failed, just as everybody else's have; but invariably the decay has been at those points where I think there was failure in accurate condensation of the filling at the margin, and it occurs more frequently at the mesio-proximal in the upper teeth than elsewhere. Perhaps the idea that I got from reading Dr. Arthur's work was different from that which others may have received. I remember especially Dr. Perry, of New York, criticising the method at one time. He described some of his cases, and how hard he had to work to restore them afterwards. Dr. Perry was personally acquainted with Dr. Arthur. Judging from his description, he secured a very different idea of the Arthur separation, by personal contact, than that which I conceived from reading. Perhaps it was my good fortune not to have seen Dr. Arthur, and not to have taken what was perhaps so extravagant a course. My knowledge of it and conception were diluted by successive cultures from Baltimore away down to Maine, so, instead of the full small-pox, I had only varioloid.

On the molars and bicuspids, of course, it would not now be used to any such extent as Dr. Arthur advised; and I think in Dr. Perry's cases it was that class of cavities that he was referring to. But I am very sure, even on these cases, if you trim back the edges a little when commencing the operation, as Dr. Arthur recommends, you will find it gives good access to the cavities, after which the edges may be cut until sound margin is obtained; then, properly contouring the teeth and getting the point of contact, you have all that is obtained by wide separation and extensive cutting away for prevention.

And I must disagree a little with the statement that dentine, if left exposed, necessarily decays. I was taught many years ago that if dentine was exposed and well polished, and not left in contact, it was about as little likely to decay as enamel itself. A number of years ago I had a young man in my chair who had had his front teeth treated in Germany for caries. A file, thick enough to remove the decay, had been passed between the incisors up nearly to the gingival margin, but not quite, leaving a shoulder, so that they were held apart. The filling had been done, he told me, twelve years before, and when I saw him there had been no sign of recurrence of decay.

It is very easy to be extravagant in our conceptions; it is very easy to be extravagant in our manipulations; and it is very easy to be led by our enthusiasm to feel that this one way is *the* way, and the only way, and that, consequently, every other way must be a failure. Now, I do not think that is the principle for us to go on, because I think it is not true. I was remarking this to-day at a meeting of our instructors. We have some dozen or fifteen different teachers there in operative dentistry, and I presume there are as many individual ways of doing the operations as there are individuals there. I take it for granted that every one of them is right, because any man who has a plan in his mind works out a system, and while there will be some peculiarities about it, if it is well done, it surely will be a success.

I know that our good friend from St. Paul here is a success according to the plan that he has worked out for himself.

*Dr. Brackett.*—Mr. President, my views are pretty well known. I have been very much interested in the essay, and I have listened with cordial assent to nearly all that has been said.

I had a little experience in my earlier years of practice with the Arthur method. It was brought into the school with something of a spirit of enthusiasm; and I used it in a few cases in my own practice afterwards. In the use of the Arthur method I never went very far. Early in my practice I got the idea that wedging in advance and making contour fillings was by far the best plan for the patient's comfort in mastication. If this method of filling is capably carried out, there is not only avoidance of the annoyance of the lodgement of food between the teeth and its extremely uncomfortable impingement against the gum, but the liability to new decay coming about the filling is very greatly lessened. So I have been practically all along a believer in advance wedging and full restoration, or exaggeration, of contour.

I believe, also, as has been suggested by the last speaker, that there are a great many instances of undue wedging, making more space than is necessary. Four thicknesses of tape, as is asked for by some operators, seem to me more than is necessary for making, with properly shaped instruments, fillings that may fully restore the contour.

I have been very much impressed by what Dr. Bogue has said to us about how many of our embarrassments may be traced back to a loss of natural conditions. One of the instances where I have lamentably failed to give comfort by restoration or exaggeration of the proximal contour has been in the case where teeth have been lost, and the remaining teeth, perhaps of people past middle life, are not solid in their sockets. Notwithstanding the contour is restored and exaggerated, patients complain of the nuisance of food being driven into the spaces. This is on account of the teeth springing in their sockets, or moving away from each other, being permitted to do so by the breaks in the arch.

Dr. Bogue has gone very far in answering one question which I had in mind of the very great desirability of not lessening the number of teeth which nature has put in the mouth, that they may be kept shoulder to shoulder for the advantage of contact.

On one point I cannot agree with the essayist. If I rightly understood him, he expressed himself to the effect that, if the ideal operation cannot be performed, we should not undertake to operate at all. (Dr. Wedelstaedt: "Unless lasting benefit will result.") If the essayist had said that, I would suggest that all of us see cases where, if we are to render the patient service at all, we have to depart somewhat from following any one plan or operating upon any one particular principle. There are instances in the practice of all of us where the advance wedging and the contour filling, or its exaggeration, for one reason or another, are not advisable. I think every operator should be a man of resourcefulness. I think he should be a man of sufficient comprehensiveness of ideas not to be limited to any one line of operating. The principal filling that I made this morning was in a case where there had been more than a full restoration of contour, which had not been a success. The teeth were not very solid in their sockets, and there had been continued trouble with the lodgement of food until the filling fell out. I filled the cavity, leaving a wide space, at the patient's earnest request, and in accordance with my own conviction that that was the best thing to do under the circumstances.

Varying circumstances require varying resources. I do not think we should decline to operate in any case, even if the ideal result is not to be obtained, if we are conscientiously impressed that a plan which we may adopt will render the patient the best service that the condition permit. We are accustomed with many things in our experience to say that *if* the circumstances were so and so, consequences would be so and so, or our procedure would be so and so; but practically that "if" is not the right thing to put there. It is a condition that confronts us, and we must take that condition, or take the group of conditions, as they are actually, and do with those existing conditions the best which our ability and the circumstances permit.

*Dr. Bogue.*—Mr. President, in answer to the question, I am perfectly willing to tell Dr. Wedelstaedt how to separate teeth, and incidentally will say to the rest of the gentlemen that if, by using what is called Japanese grass-line, a loop is put between two teeth, from the outside in, and then the two free ends of the loop are tied into that loop between the teeth with two or three knots, it ties the silk in such fashion that you only have to leave it for two or three or four days, and the work is done. The loop is drawn through between the teeth by using a piece of floss-silk to draw it through at the gum margin.

*Dr. Wedelstaedt.*—Allow me, first of all, to thank you for the kind words you have spoken about me and the work I am doing for the profession. I do what I do because I love my work. Permit me to tell you a story which will answer one of the gentlemen much better than anything else that I know of.

"An old Scotchman lived far out in the country and way in the interior. He had a son who lived with him until he was nineteen years of age. His father then took him into the city. The boy did not sleep very well, and at daybreak he was up and wandering around in the yard, which was back of the house where they lodged. Immediately back of the yard was a railway track. While the boy was wandering around the yard, the limited express went by. Never having seen a train of cars before, he looked at it a moment, and then ran into the house and up to his father, who was still asleep. Frantically grabbing his father, he said, 'Father! father! gie right up; gie right up; the smithy has run away with a whole row of houses, and is now at the lower end of the town!'"

Now, what was the cause of this state of affairs? Simply the lad's environment and surroundings. I do not think it is necessary for me to say anything more. This fully answers the gentleman.

I am indeed pleased to have been able to have met with you, and I thank you for the cordial reception that you have given my essay.

On motion, adjourned.

CHARLES H. TAFT, D.M.D.,
*Editor American Academy of Dental Science.*

